# Chromosome-specific polymorphic SSR markers in tropical eucalypt species using low coverage whole genome sequences: systematic characterization and validation

**DOI:** 10.5808/gi.21031

**Published:** 2021-09-30

**Authors:** Maheswari Patturaj, Aiswarya Munusamy, Nithishkumar Kannan, Ulaganathan Kandasamy, Yasodha Ramasamy

**Affiliations:** 1 Institute of Forest Genetics and Tree Breeding, Coimbatore 641002, India; 2 Centre for Plant Molecular Biology, Osmania University, Hyderabad 500007, India

**Keywords:** *Eucalyptus camaldulensis*, *E. grandis*, *E. tereticornis*, simple sequence repeats, whole genome resequencing

## Abstract

*Eucalyptus* is one of the major plantation species with wide variety of industrial uses. Polymorphic and informative simple sequence repeats (SSRs) have broad range of applications in genetic analysis. In this study, two individuals of *Eucalyptus tereticornis* (ET217 and ET86), one individual each from *E. camaldulensis* (EC17) and *E. grandis* (EG9) were subjected to whole genome resequencing. Low coverage (10×) genome sequencing was used to find polymorphic SSRs between the individuals. Average number of SSR loci identified was 95,513 and the density of SSRs per Mb was from 157.39 in EG9 to 155.08 in EC17. Among all the SSRs detected, the most abundant repeat motifs were di-nucleotide (59.6%–62.5%), followed by tri- (23.7%–27.2%), tetra- (5.2%–5.6%), penta- (5.0%–5.3%), and hexa-nucleotide (2.7%–2.9%). The predominant SSR motif units were AG/CT and AAG/TTC. Computational genome analysis predicted the SSR length variations between the individuals and identified the gene functions of SSR containing sequences. Selected subset of polymorphic markers was validated in a full-sib family of eucalypts. Additionally, genome-wide characterization of single nucleotide polymorphisms, InDels and transcriptional regulators were carried out. These variations will find their utility in genome-wide association studies as well as understanding of molecular mechanisms involved in key economic traits. The genomic resources generated in this study would provide an impetus to integrate genomics in marker-trait associations and breeding of tropical eucalypts.

## Introduction

*Eucalyptus* belongs to the family Myrtaceae, cultivated throughout the tropical and subtropical regions of the world. It is considered to be a major raw material for paper industry and has interesting potential in wood panel, solid wood, charcoal, biofuel and pharmaceutical sectors. Leaf extracts present a wide range of phenolic compounds having antioxidant effects [1], while its bioactive metabolites have demonstrated several ethnopharmacological properties [2]. Genetic improvement programs for limited number of eucalypt species have been implemented across many countries including India, South Africa, China, Brazil, Thailand and Australia. Owing to their great economic value, species-specific genetic and genome resources are progressively increasing. Some of the species like *E. grandis*, *E. camaldulensis*, and *E. tereticornis* are highly significant for the tropical countries because of their unique properties in paper pulp production and abiotic stress tolerance [3,4]. These species are predominantly used in inter-specific hybridizations, where hybrid breeding strategy is always employed to combine the traits of interest and realize the genetic gains [5].

Genetic marker resources such as simple sequence repeats (SSR) and single nucleotide polymorphisms (SNPs) have been used as powerful tools for identification of individuals, analysis of population structure and genetic diversity, DNA fingerprinting, genetic mapping and localization of QTLs, marker‐assisted selection and genomic selection [6]. SSRs are the popular genetic markers because of their abundance, ubiquitous distribution, high polymorphism, codominant inheritance, multiallelism and ease of assay by PCR [7]. Numerous genomic and EST‐derived SSR markers have been reported in *Eucalyptus* [8-10]. SSRs can be cross-transferred between closely related species but success rate of intra-genus transferability in eucalypts varied from 40% to 96% [6]. However, identification of polymorphic SSRs between closely related individuals is often difficult because of its genome synteny and colinearity across the species [11] warranting large scale development of SSR markers having polymorphism between individuals.

Latest advances in sequencing technology and bioinformatic research have provided an unparalleled opportunity to identify high-quality, cost and time-effective polymorphic SSR markers in several plant species [12,13]. Further, continuing decrease in the cost of genome sequencing unfolded possibilities for massive identification of polymorphic SSRs as well as large scale genotyping [14]. Moreover, in the species with known genome sequence information, whole genome resequencing strategy is employed to extract polymorphic SSRs rapidly. Mapping parents and segregating populations of *Raphanus sativus* were resequenced at whole genome level and genetic map was constructed with polymorphic SNPs, SSRs, and InDels [15]. Whole genome resequencing was adopted for the development of polymorphic SSR markers between Chinese oriental melon and Korean oriental melon, and many thousands of SSRs, SNPs, and InDels were identified [16]. In *Nicotiana tabacum* whole genome resequencing was carried out in two genotypes to comparatively analyse SSR variations and identify SNPs, InDels, structural variations, and copy number variations for generation of more number of genetic markers [17]. In *Liriodendron chinense*, four genotypes were sequenced at low coverage scale and identified genome-wide SSRs, SNPs, and InDels to assist in molecular genetics, genotype identification, genetic mapping, and molecular breeding [18]. Several thousands of SSR markers were identified for *Ensete ventricosum* by analysing the genome sequence data of four landraces and *in silico* methods were adopted for the development of polymorphic markers [19]. Computational tools such as GMATA [20], PolyMorphPredict [21], SSRgenotyper [22], and MultiplexSSR [23] facilitate SSR genotype calling from resequenced data of individuals within natural populations, germplasm collections and segregating biparental mapping populations.

Accordingly, in the present study, low depth whole genome resequencing was carried out in four genotypes of three tropical species of *Eucalyptus* namely *E. grandis*, *E. camaldulensis* and *E. tereticornis*. The objectives of the study were to (1) characterize the SSR markers on different chromosomes, motif types, frequency and length distribution, (2) *in silico* identification of polymorphic SSR primers between the selected eucalypt species, functional annotation and design candidate primer pairs, (3) validate a subset of SSR primers by PCR amplification in a full-sib family of eucalypts. The distribution of SSR polymorphisms among selected individuals is discussed in relation to their application in cost-efficient genotyping of mapping populations. In eucalypt breeding programs, these markers are regarded as valuable genetic reservoir for genotype identification, genetic diversity analysis, hybrid purity testing and marker assisted selection.

## Methods

### Plant material and DNA isolation

Four eucalypt clonal accessions, *E. tereticornis* (ET217), *E. camaldulensis* (EC17), *E. tereticornis* (ET86), and *E. grandis* (EG9) were selected for low depth whole genome resequencing. These accessions have been frequently used as the parents for inter-specific full-sib cross generation (ET217 × EC17; ET86 × EG9). SSR polymorphism validation experiments were conducted with 80 individuals of a full-sib family, ET86 × EG9. Juvenile leaves were used for total genomic DNA isolation with DNeasy plant DNA mini kit (Qiagen Inc., Valencia, CA, USA) as per the manufacturer’s instructions.

### DNA quality analysis and whole genome sequencing

Quality of the DNA was checked on 0.8% Agarose (A9539, Sigma-Aldrich, St. Louis, MO, USA) gel at 120 V for approximately 60 min or until the samples reached 3/4th of the gel. Absorbance ratio at 260/280 was measured with a NanoDrop 2000 UV-Vis spectrophotometer (Thermo Fisher Scientific, Waltham, MA, USA). A Qubit 2.0 Fluorometer (Q32866, Invitrogen, Carlsbad, CA, USA) was used with a Qubit dsDNA HS Assay Kit (Q32854) to confirm DNA input of 10 μg before shearing. All the DNA samples passed the QC were subjected to paired-end sequencing library preparation with NEB Ultra DNA library preparation kit (New England BioLabs, Ipswich, MA, USA). The quantity and quality check of library was carried out using Agilent TapeStation 2200 System (Agilent Technologies, Santa Clara, CA, USA). Whole genome sequencing of the four eucalypt samples was performed by AgriGenome Labs Pvt Ltd, Hyderabad on an Illumina HiSeq 4000/X ten Genome Analyzer using 2 × 150 bp chemistry. The fastq files were pre-processed using AdapterRemoval2 (v2.2, default parameters). The raw reads were checked for presence of adapter sequences and reads that’s average quality score less than 30 (<30 phred score) in any of the paired-end reads were filtered out.

### Genome annotation and analysis

Preliminary analysis was carried out to construct reference-based assembly for each of the four samples, its cleaned reads were aligned against the reference genome *Eucalyptus grandis* v2.0 (11 chromosomes) downloaded from Phytozome (http://phytozome.jgi.doe.gov/pz/portal.html). The short read sequences were assembled into 11 pseudomolecules for each individual. Only uniquely mapped reads were considered for pseudomolecule development. The reads were aligned using BWA (v0.7.17-r1188) individually to the reference genome and reference guided consensus assemblies were generated for each individual using samtools/bcftools suite (v1.9) in FASTA format for further analysis.

An online integrated genome sequence annotation pipeline GenSAS v6.0 [24] was used to annotate the pseudomolecules of four *Eucalyptus* individuals. GenSAS v6.0 was used for various analyses such as repeat masking, gene prediction, annotated gene models and mapping of predicted proteins. Repeats in the pseudomolecules were masked via RepeatMasker v.4.0.7 and RepeatModeler using *Arabidopsis thaliana* as reference. Genes were predicted using the *ab initio* tools and Augustus v.3.3.1 using models from *Arabidopsis thaliana*. Augustus was run using gene models from *Arabidopsis*, finding genes on both strands, and allowing partial models. Sequence alignments were performed using BLAST, BLAT, and PASA against NCBI plant RefSeq database. Multiple lines of evidence were integrated into a gene consensus using EVidenceModeler with default weights. Predicted proteins were compared to the NCBI plant RefSeq database and SwissProt using BLASTP. Protein families were classified using the InterPro database and InterProScan v. 5.8-49.0. An estimate of the completeness of the predicted proteins was calculated using the program BUSCO v. 3.0.2. The GO-Slim and Enzyme-code annotation were performed using Blast2GO for the predicted proteins. Pathway annotation was conducted by mapping the sequences obtained from Blast2GO to the contents of the Kyoto Encyclopedia of Gene and Genomes Automatic Annotation Server (KAAS; http://www.genome.jp/kegg/kaas/ (1 April 2020, date last accessed). The Venn diagrams were generated using jvenn to differentiate common genes across individuals [25]. Transcription factors, transcriptional regulators and protein kinases were identified and classified into different families using the iTAK pipeline v1.7 [26].

### Genome-wide SNP and InDel detection

Genome-wide SNPs and InDels were analysed in the four *Eucalyptus* genomes using reference-based assembly of *E. grandis* to document the genetic variants. The reference genome was indexed and the mapping was done using Bowtie2 Aligner [27]. SAMtools was used to convert the generated SAM file to BAM format. The BAM file was sorted and indexed. The reference was also indexed using faidx command of SAM tools [28]. The sorted BAM file was used to generate BCF file using mpileup command of the same package. The variant calling was conducted using bcftools by converting the BCF file to VCF with parameters such as low quality filter >20 and DP >100.

### Identification of SSRs and detection of polymorphism

FASTA formatted pseudomolecules of *Eucalyptus* were analyzed for frequency and density of SSRs using the Perl script MIcroSAtelitte (MISA; http://pgrc.ipk-gatersleben.de/misa/). Initially SSRs of 2–6 nucleotides motifs were identified with the minimum repeat unit defined as 10 for mono-nucleotides, 7 for di-nucleotides, 5 for tri-and tetra-nucleotides, and four each for penta- and hexa-nucleotides. Compound SSRs were defined as ≥2 SSRs interrupted by ≤100 bases. SSR length was classified into three categories in accordance with repeat lengths as less than 20 bp (<20), 20-40 bp, and above 40 bp (>40). Microsatellites located on the 11 pseudomolecules were used to amplify the genomic sequences of ET86 × EG9 and ET217 × EC17 employing the ePCR module of GMATA software [20]. The primer nucleotide mismatch allowed was no more than one nucleotide and other parameters were set as default. The polymorphic primers were selected based on difference in number of repeat units present in between the genomes of ET86 × EG9 and ET217 × EC17 and polymorphic information content value greater than 0.3 to ensure the SSR polymorphism.

### Genotyping of a full-sib family

A subset of 58 primer pairs which were polymorphic *in silico* in the cross ET86 × EG9 was randomly selected for validating the SSR loci amplification in the 80 full-sib progenies. PCR amplification was performed following the protocol of [29] and the products were separated on 7% polyacrylamide gel electrophoresis. The gel was run at 220 V constant power for 3 h and bands visualised by standard silver staining methods. Allele size variations were measured with Alpha Ease FC 5 software (Alpha Innotech, San Leandro, CA, USA).

## Results and Discussion

### Annotation of genes and repetitive elements

In the present study, four individuals belonging to *E. grandis*, *E. camaldulensis*, and *E. tereticornis* were subjected to short read sequencing with approximate of genome coverage of 10× for each genotype. The final assemblies had a total length of 611.8 to 612.2 Mb (Table 1), and each assembly was arranged into 11 pseudomolecules and deposited in NCBI (Biosample SAMN14826404, SAMN14826405, SAMN14826406, and SAMN14826407). Size of the individual pseudomolecule varied between 37.7 and 83.9 Mb, with an average of 55.6 Mb. The percentage of genes identified in the *Eucalyptus* genome sequence showed that nearly 82%‒85% of the genome was represented (Table 2). The results were in accordance with *E. pauciflora*, where the BUSCO genes varied from 70.5%‒91.3% in different assemblies [21].

**Fig. 1.**
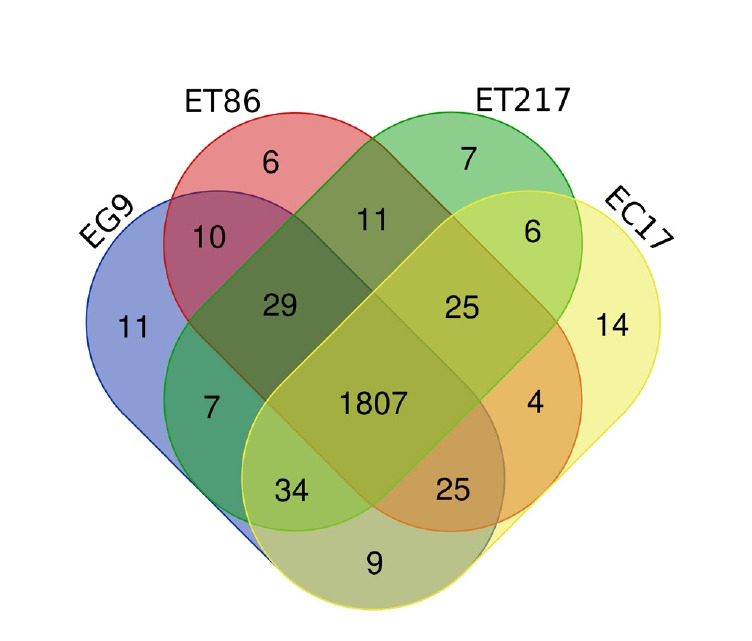
Venn diagram shows the number of shared and unique gene families among the four *Eucalyptus* individual analyzed. Each color represents one individual (*E. camaldulensis* [EC17], *E. tereticornis* [ET86 and ET217], and *E. grandis* [EG9]).

**Fig. 2.**
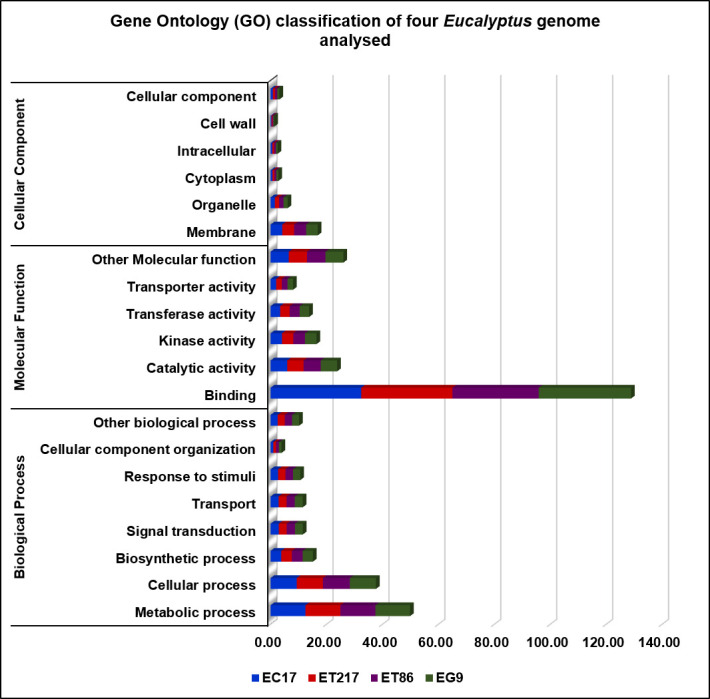
Gene ontology classification of four *Eucalyptus* genome analyzed (*E. camaldulensis* [EC17], *E. tereticornis* [ET86 and ET217], and *E. grandis* [EG9]) for biological process, cellular component, and molecular function.

Repeats in the genome of four *Eucalyptus* individuals were identified and masked which comprised to maximum 53.65 % (ET86) and minimum 35.82% (ET217) of the assemblies (Supplementary Table 1). Unclassified repeats occupied the maximum amount of genome repeats totalling to 64.57, 51.04, 48.16, and 43.56% of the ET86, EC17, EG9 and ET217, respectively. The LTR elements including Gypsy and Copia repeats had occupied next highest type of repeat classes across the individuals analysed. Gene prediction with NCBI RefSeq resulted in maximum of 57,075 (EG9) to minimum of 49,515 (ET86) protein-coding sequences (Table 3). Out of 2,005 gene families analysed 1,807 common genes were identified, accounting for 90.0% of the total protein-predicted genes highlighting the close relationship among the species (Fig. 1). Very limited number of genes was found to be species-specific, some of the unique genes such as disease resistance protein RPS4 and chitin elicitor receptor kinase 1 involved in defense activation were identified in ET86 and EC17, respectively. Gene ontology classification revealed higher proportion of genes related to molecular function followed by biological process, and cellular components (Fig. 2). Analysis of transcription factors (TFs), transcriptional regulators, and protein kinases (PKs) identified an average of 1,807 (from 69 families), 393 (from 24 families), and 2,137 (from 120 families) genes respectively in the four genomes analysed (Supplementary Table 2). Further, the eucalypt genome encoded majority of PKs belong to the receptor-like kinase family.

The reference assemblies generated in this study were primarily in accordance with the *Eucalyptus* annotation release 101 (https://www.ncbi.nlm.nih.gov/genome/annotation_euk/Eucalyptus_grandis/101/). The released assembly of *E. grandis* had totally 55,643 genes belonging to various classes like protein coding, non-coding, pseudogenes and genes with variants [30]. Recent studies in eucalypts highlighted the role of TFs and PKs associated with secondary cell wall development [31], biotic resistance [32] and abiotic tolerance [33,34]. Accordingly, results of this study offer a comprehensive view of regulatory sequences associated with almost all essential cellular functions and provides a foundation for further characterization.

### Identification of SNPs and InDels

Sequences mapped to the assembled chromosomes were analysed to predict the putative SNPs and InDels (Supplementary Table 3). The number of SNPs recorded across the four eucalypt genomes analysed was 727,996 (EG09), 1,225,836 (ET86), 1,207,912 (ET217), and 1,170,967 (EC17). Similarly, the InDels observed were 104,542 (EG09), 141,591 (ET86), 142,384 (ET217), and 134,986 (EC17). Maximum number of SNPs and InDels were recorded in longer pseudomolecules such as 03, 08, and 05. In the recent past, genome-wide association and genomic selection approaches were implemented using SNPs and InDels in eucalypt species [35-37].

### SSR distribution and polymorphism

SSRs were detected using MISA in assembled pseudomolecules and SSR prediction statistics are presented in Table 4 and 5. Average number of SSR loci identified was 95,513 and their distribution across species were 94,889 (EC17), 95,373 (ET217), 95,425 (ET86), and 96,365 (EG9), respectively. The number of SSRs was found to be correlated with the chromosome length. Longer chromosomes like 03, 05 and 08 had more than 11,000 SSRs whereas shorter ones like 04, 09 and 10 had lower number of SSRs (Table 6). The highest frequency of the grouped SSR motif units was dimer AG/CT (44.2%‒46.9%) and among the tri, tetra, penta and hexamers AAG/TTC, ACAT/ATGT, AAAAT/ATTTT and AAAAAG/CTTTTT was most common, respectively (Supplementary Table 4). Tri-nucleotide motif type AAG/TTC was reported to be the most common in eucalypts and in many other dicot plants such as *Arachis*, cucumber, soybean, *Arabidopsis* and grape [38,39]. Among the 17 different tri-nucleotides ACA/TGT was the least commonly present motif type. The predominant repeat motif types are in accordance with earlier reports on eucalypts [38,40]. SSR class with the length of <20 bp was more abundant followed by 20-40 bp and >40 bp in all the four individuals analysed (Supplementary Table 5).

**Fig. 3.**
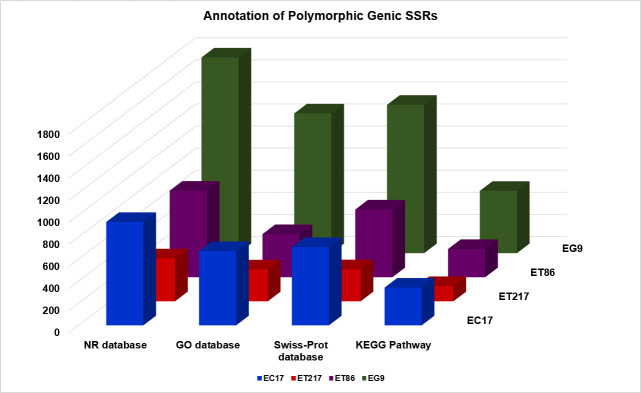
Annotation of polymorphic genic simple sequence repeats associated to the RefSeq non-redundant (NR), Gene Ontology (GO), SwissProt, and Kyoto Encyclopedia of Gene and Genomes (KEGG) database (*E. camaldulensis* [EC17], *E. tereticornis* [ET86 and ET217], and *E. grandis* [EG9]).

*Eucalyptus* species are closely related with overlapping geographical locations having high amount of gene flow among species [41], thus pose difficulty in choosing polymorphic SSRs. In this study, SSR polymorphism in perfect repeat motifs was determined by *in silico* characterization of SSR length variation between the parents of the cross, ET217 × EC17 and ET86 × EG9. The cross, ET217 × EC17, had an average of 95,131 SSRs of which 13.4% (12,725) markers from pseudomolecule 1, 8 and 10 were showing polymorphism. In the case of ET86 × EG9, all the chromosomes harboured polymorphic SSRs except 3, 6, 9, and 11. Although the cross had an average of 95,895 SSRs, only 25.7% (24,688) of the SSRs could be converted into usable markers. The number of genic SSRs which were polymorphic among the clonal accessions analysed are shown in Fig. 3. In both the crosses, among the five repeat motif types, the di-nucleotide showed maximum polymorphism (76.0 %) followed by tri-nucleotides (18.0%). The *in silico* polymorphic markers can be utilized not only for high density genetic linkage map generation but also for variety of purposes including genome-wide marker-trait associations and population genetic studies.

### Validation of polymorphic SSR markers

**Fig. 4.**
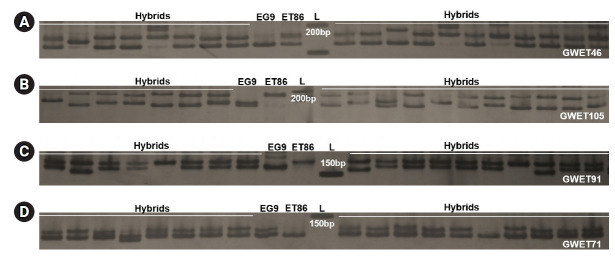
Gel images of simple sequence repeat markers with primers GWET 46 (A), GWET 105 (B), GWET 91 (C), and GWET 71 (D) for the cross ET86 × EG9. Hybrids, full-sib progenies of ET86 × EG9; ET86, *E. tereticornis*; EG9, *E. grandis*; L, 50 bp DNA ladder.

Among the 58 primer pairs, 46 (81%) successfully amplified in 80 full-sib progenies of the cross ET86 × EG9 and 12 (19%) failed to generate the PCR products. Out of 46 primer pairs, 35 (62%) generated one or two polymorphic alleles and remaining 11 showed monomorphic products (Fig. 4, Supplementary Table 6). A similar study in two closely related species *Capsicum chinense* and *C. annuum* employed *in silico* produced SSRs to ascertain their effectiveness and 71.2% polymorphism was recorded [42]. In the full-sib family of *Trachinotus ovatus*, a computational pipeline Multiplex SSR was employed to predict polymorphism and 85% success in PCR amplification was recorded [23]. Further, these results confirmed that predicted SSRs polymorphism can be utilized for accurate genotyping in capillary based systems economically and efficiently.

### Conclusion

Experiments on the whole genome resequencing are becoming increasingly frequent, and eucalypts are no exception. The majority of resequencing experiments were able to detect significant genetic variations between the sequenced accessions and the reference genome. In the genetic advancement of eucalypts, genome-enabled approaches have become indispensable. Some of the examples include integration of DNA markers in commercial breeding of eucalypts by paper industries for quality control in clonal forestry and hybrid purity. International research consortia are using genomics to identify chromosomal locations governing commercially important traits. Abundant SSRs have been discovered in the released genomes of *E. grandis*, *E. camaldulensis*, and *E. pauciflora*. However, the inherent drawbacks associated with identification of polymorphic SSRs for mapping population genotyping, varietal fingerprinting and population genomics continue to exist. Affordable whole genome resequencing technology along with appropriate bioinformatics tools makes the prediction of SSR variations across highly similar genomes possible. The findings of this study improved our knowledge on genetic variations between eucalypt individuals and developed pre-screened SSR markers to genotype the mapping populations. Further, a large set of chromosome anchored markers and TFs were discovered. The SNPs generated would find its application in high density genetic linkage map generation. It forms a valuable genomic resource with promising applications in QTL based selection and breeding, genomic selection, conservation of genetic resources and improvement of eucalypt germplasm.

## Figures and Tables

**Table 1.** Description of sequence data generated for four *Eucalyptus* individuals

Sample ID	No. of raw reads	No. of bases (Mb)	GC percent	% Squences with Q30	Genome assembly size (Mb)
*E.camaldulensis* (EC17)	50,810,080	7,621.5	41.2	91.1	611.9
*E.tereticornis* (ET217)	41,507,796	6,226.2	38.8	89.5	611.9
*E.tereticornis* (ET86)	43,514,058	6,527.1	40.0	90.3	611.9
*E.grandis* (EG9)	46,840,164	7,026.0	39.6	90.3	612.3
Average	45,668,025	6,850.2	40.0	90.0	612.0

**Table 2.** Summary of BUSCO analysis results for the four *Eucalyptus* assemblies

BUSCO assessment	*E. camaldulensis* (EC17)	*E. tereticornis* (ET217)	*E. tereticornis* (ET86)	*E. grandis* (EG9)
Complete BUSCOs (C)	1,206 (83.8)	1,211 (84.1)	1,181 (82.1)	1,219 (84.7)
Complete and single-copy BUSCOs (S)	1,141 (79.2)	1,162 (80.7)	1,124 (78.1)	1,153 (80.1)
Complete and duplicated BUSCOs (D)	65 (4.5)	49 (3.4)	57 (4.0)	66 (4.6)
Fragmented BUSCOs (F)	114 (7.9)	99 (6.9)	115 (8.0)	99 (6.9)
Missing BUSCOs (M)	120 (8.3)	130 (9.0)	144 (9.9)	122 (8.4)
Total BUSCO groups searched	1,440 (100)	1,440 (100)	1,440 (100)	1,440 (100)

Values are presented as number (%).

**Table 3.** Descriptive details on genome annotation of four *Eucalyptus* individuals

Sample ID	NCBI RefSeq proteins					No. of predicted proteins with Swiss-Prot database	No. of proteins annotated for functionality
	Total	Less than 100 amino acids	Percent similarity to *Eucalyptus*	Uncharacterized protein	Maximum and minimum amino acid length		
*E. camaldulensis* (EC17)	54,656	7,231	90.9	17,991	5,164 : 34	30,892	10,514
*E. tereticornis* (ET217)	54,852	7,415	91.1	21,251	5,422 : 34	28,067	10,665
*E. tereticornis* (ET86)	49,515	6,492	94.1	16,789	5,372 : 34	27,148	10,424
*E. grandis* (EG9)	57,075	7,333	90.6	22,985	5,904 : 34	31,807	10,753

**Table 4.** Distribution of SSRs in *Eucalyptus* genome

Parameter	*E. camaldulensis* (EC17)	*E. tereticornis* (ET217)	*E. tereticornis* (ET86)	*E. grandis* (EG9)
Total No. of SSR loci	94,889	95,373	95,425	96,365
Loci distance (kb)	6.45	6.42	6.41	6.35
Density (SSRs/Mb)	155.08	155.87	155.96	157.39
SSR length <20 bp	86,824	87,628	87,585	88,719
SSR length 20‒40 bp	7,649	7,326	7,421	7,222
SSR length >40 bp	416	419	419	424

SSR, simple sequence repeat.

**Table 5.** Different types of SSR loci identified in four *Eucalyptus* individuals

SSR type (%)/Sample ID	*E. camaldulensis* (EC17)	*E. tereticornis* (ET217)	*E. tereticornis* (ET86)	*E. grandis* (EG9)
Di-nucleotide	62.5	59.9	59.9	59.6
Tri-nucleotide	23.7	27.0	27.0	27.2
Tetra-nucleotide	5.6	5.3	5.2	5.3
Penta-nucleotide	5.3	5.0	5.0	5.2
Hexa-nucleotide	2.9	2.7	2.8	2.8

SSR, simple sequence repeat.

**Table 6.** Various SSR types and their distribution among the 11 pseudomolecules of the four *Eucalyptus* clonal accessions

SSR type/Chromosome	1	2	3	4	5	7	8	9	10	11
*E. camaldulensis* (EC17)										
Di-nucleotide	4,389	5,543	6,839	3,862	6,588	5,275	6,946	3,606	3,888	4,495
Tri-nucleotide	1,916	2,518	3,063	1,656	2,796	2,219	3,169	1,679	1,840	2,029
Tetra-nucleotide	396	409	624	351	647	430	673	329	350	385
Penta-nucleotide	374	432	639	340	534	446	582	326	322	361
Hexa-nucleotide	192	256	325	159	272	222	347	182	192	217
*E. tereticornis *(ET217)										
Di-nucleotide	4,440	5,600	6,878	3,880	6,614	5,278	6,870	3,615	3,952	4,499
Tri-nucleotide	1,940	2,565	3,085	1,704	2,855	2,260	3,229	1,708	1,898	2,031
Tetra-nucleotide	390	430	665	346	646	435	659	332	342	380
Penta-nucleotide	380	427	630	339	528	439	580	317	319	352
Hexa-nucleotide	187	262	295	160	291	226	335	193	179	197
*E. tereticornis* (ET86)										
Di-nucleotide	4,442	5,590	6,919	3,882	6,577	5,274	6,922	3,653	3,937	4,471
Tri-nucleotide	1,954	2,552	3,086	1,687	2,869	2,255	3,206	1,705	1,888	2,021
Tetra-nucleotide	371	420	648	351	668	458	677	330	348	365
Penta-nucleotide	377	432	647	345	508	438	579	330	326	371
Hexa-nucleotide	181	265	302	156	294	244	313	195	176	228
*E. grandis* (EG9)										
Di-nucleotide	4,429	5,545	6,877	3,950	6,653	5,253	6,925	3,665	4,003	4,521
Tri-nucleotide	1,984	2,567	3,202	1,759	2,893	2,317	3,265	1,723	1,879	2,005
Tetra-nucleotide	401	411	665	367	644	453	700	327	333	356
Penta-nucleotide	406	442	652	348	548	440	582	344	340	384
Hexa-nucleotide	182	285	310	173	297	225	339	197	178	222

SSR, simple sequence repeat.

## Supplementary Materials

Supplemental Table 1. Repeat elements present in clonal accessions of *Eucalyptus* (*E. camaldulensis* (EC17), *E. tereticornis* (ET86 and ET217), *E. grandis* (EG9)

Supplemental Table 2. Transcription factors (TFs), transcriptional regulators (TRs), and protein kinases (PKs) predicted in the clonal accessions of *Eucalyptus* (*E. camaldulensis* (EC17), *E. tereticornis* (ET217 and ET86), *E. grandis* (EG9)

Supplemental Table 3. SNPs and InDels predicted in clonal accessions of *Eucalyptus* (*E. camaldulensis* [EC17], *E. tereticornis* [ET217 and ET86], *E. grandis* [EG9])

Supplemental Table 4. Top 15 types of simple sequence repeat motifs in clonal accessions of *Eucalyptus* having 1% and above distribution (*E. camaldulensis* (EC17), *E. tereticornis* (ET217 and ET86), *E. grandis* (EG9)

Supplemental Table 5. Length distribution of top 15 types of simple sequence repeat motifs in clonal accessions of *Eucalyptus* (*E. camaldulensis* (EC17), *E. tereticornis* (ET217 and ET86), *E. grandis* (EG9)

Supplemental Table 6. SSR marker information and details on primer pairs synthesised for validation in the cross *E. tereticornis* ET86 x *E. grandis* EG9.
