# Supplementary material for: Chromosome-specific polymorphic SSR markers in tropical eucalypt species using low coverage whole genome sequences: systematic characterization and validation
Source: Genomics Inform. 2021 Sep 30;19(3):e33. doi: 10.5808/gi.21031 (PMC8510864; doi:10.5808/gi.21031)
Supplement: Supplemental Table 1. — Repeat elements present in clonal accessions of Eucalyptus (E. camaldulensis (EC17), E. tereticornis (ET86 and ET217), E. grandis (EG9) [file gi-21031suppl1.pdf]

**Supplementary Table 1.** Repeat elements present in clonal accessions of *Eucalyptus* (*E. camaldulensis* (EC17), *E. tereticornis* (ET86 and ET217), *E. grandis* (EG9)

| Repeat type | EC17 | | | ET86 | | | ET217 | | | EG9 | | |
|---|---|---|---|---|---|---|---|---|---|---|---|---|
| | Copy number | Total (bp) | % | Copy number | Total (bp) | % | Copy number | Total (bp) | % | Copy number | Total (bp) | % |
| LINE | 12,010 | 13,286,007 | 3.67 | 15,598 | 10,036,488 | 3.54 | 11,930 | 12,908,797 | 3.29 | 12,457 | 14,070,671 | 3.80 |
| LTR elements | 56,594 | 73,892,165 | 20.41 | 62,821 | 44,747,216 | 15.78 | 83,502 | 102,694,206 | 26.15 | 64,667 | 80,472,238 | 21.72 |
| SINE | 0 | 0 | 0.00 | 63 | 5,642 | 0.00 | 141 | 28,070 | 0.01 | 0 | 0 | 0.00 |
| DNA/hAT-Ac | 813 | 546,213 | 0.15 | 932 | 486,396 | 0.17 | 1,023 | 1,034,873 | 0.26 | 1,349 | 573,002 | 0.15 |
| DNA/hAT-Tag1 | 191 | 99,953 | 0.03 | 183 | 152,361 | 0.05 | 227 | 104,335 | 0.03 | 150 | 95,003 | 0.03 |
| DNA/MuLE-MuDR | 1,042 | 1,323,175 | 0.37 | 2,192 | 1,489,172 | 0.53 | 2,206 | 1,888,827 | 0.48 | 1,927 | 1,627,857 | 0.44 |
| DNA/PIF-Harbinger | 1,847 | 1,005,024 | 0.28 | 1,081 | 562,110 | 0.20 | 995 | 535,691 | 0.14 | 778 | 961,162 | 0.26 |
| LINE/L1 | 11,793 | 13,236,304 | 3.66 | 15,598 | 10,036,488 | 3.54 | 11,569 | 12,744,080 | 3.25 | 11,961 | 13,821,606 | 3.73 |
| LTR/Caulimovirus | 2,324 | 2,996,502 | 0.83 | 3,141 | 110,826 | 0.04 | 2,732 | 709,232 | 0.18 | 2,805 | 3,672,335 | 0.99 |
| LTR/Copia | 38,537 | 54,320,822 | 15.00 | 41,014 | 3,767,468 | 1.33 | 1,858 | 2,778,095 | 0.71 | 38,061 | 52,853,878 | 14.27 |
| LTR/Gypsy | 15,733 | 16,574,841 | 4.58 | 18,286 | 29,093,387 | 10.26 | 67,121 | 86,221,683 | 21.96 | 23,635 | 23,894,121 | 6.45 |
| Unclassified | 553,748 | 184,785,756 | 51.04 | 589,120 | 183,098,663 | 64.57 | 545,398 | 171,038,474 | 43.56 | 540,726 | 178,419,464 | 48.16 |
| Total genome masked (bp) | 694,632 | 362,066,762 | 40.83 | 750,029 | 283,586,217 | 53.65 | 728,702 | 392,686,363 | 35.82 | 698,516 | 370,461,337 | 39.49 |
