# Supplementary material for: Chromosome-specific polymorphic SSR markers in tropical eucalypt species using low coverage whole genome sequences: systematic characterization and validation
Source: Genomics Inform. 2021 Sep 30;19(3):e33. doi: 10.5808/gi.21031 (PMC8510864; doi:10.5808/gi.21031)
Supplement: Supplemental Table 2. — Transcription factors (TFs), transcriptional regulators (TRs), and protein kinases (PKs) predicted in the clonal accessions of Eucalyptus (E. camaldulensis (EC17), E. tereticornis (ET217 and ET86), E. grandis (EG9) [file gi-21031suppl2.pdf]

Supplementary Table 2. Transcription factors (TFs), transcriptional regulators (TRs), and protein kinases (PKs) predicted in the clonal accessions of *Eucalyptus* (*E. camaldulensis* (EC17), *E. tereticornis* (ET217 and ET86), *E. grandis* (EG9)

**TFs**	**ET86**	**ET217**	**EC17**	**EG09**
Alfin-like	4	4	5	5
AP2/ERF-AP2	12	11	12	14
AP2/ERF-ERF	137	150	147	148
AP2/ERF-RAV	3	3	5	4
B3	81	76	84	83
B3-ARF	13	17	16	16
BBR-BPC	5	5	5	5
BES1	6	6	8	7
bHLH	123	127	120	124
bZIP	60	57	59	56
C2C2-CO-like	6	8	7	7
C2C2-Dof	22	23	25	25
C2C2-GATA	22	24	24	21
C2C2-LSD	2	1	1	2
C2C2-YABBY	6	6	6	5
C2H2	110	122	117	115
C3H	44	45	46	48
CAMTA	5	4	4	5
CPP	8	10	9	11
CSD	3	3	4	2
DBB	4	3	5	7
DBP	1	1	2	2
DDT	7	6	5	7
E2F-DP	5	5	5	5
EIL	5	5	4	5
FAR1	29	35	32	37
GARP-ARR-B	8	9	8	8
GARP-G2-like	41	41	41	41
GeBP	8	7	9	10
GRAS	101	99	95	96
GRF	7	7	6	6
HB-BELL	9	8	8	8
HB-HD-ZIP	31	32	31	31
HB-KNOX	5	4	4	3
HB-other	15	15	15	17
HB-PHD	1	1	1	1
HB-WOX	7	7	7	8
HRT	1	1	1	1

HSF	27	28	30	30
LFY	0	2	2	2
LIM	7	8	6	7
LOB	35	40	42	36
MADS-MIKC	36	36	33	35
MADS-M-type	55	58	61	64
MYB	149	141	137	153
MYB-related	75	83	85	80
NAC	166	173	172	171
NF-X1	3	3	3	3
NF-YA	8	7	9	11
NF-YB	13	14	17	14
NF-YC	10	10	8	9
NOZZLE	1	1	0	0
OFP	12	13	12	13
PLATZ	16	17	19	17
RWP-RK	17	16	15	11
S1Fa-like	1	0	1	0
SAP	1	1	1	1
SBP	15	15	17	12
SRS	5	6	7	7
STAT	2	1	2	2
TCP	16	17	19	19
Tify	15	15	15	14
Trihelix	16	16	17	14
TUB	8	8	8	8
ULT	2	2	2	2
VOZ	3	3	3	3
Whirly	2	2	2	2
WRKY	78	83	78	82
zf-HD	12	11	12	11

**TRs**	**ET86**	**ET217**	**EC17**	**EG09**
ARID	9	8	10	9
AUX/IAA	22	23	23	24
Coactivator p15	4	3	4	4
GNAT	22	24	24	25
HMG	10	11	10	11
IWS1	10	7	11	12
Jumonji	15	14	13	14
LUG	3	3	3	5
MBF1	4	4	3	2

MED6	1	1	1	1
MED7	1	1	1	1
mTERF	43	43	45	46
Others	82	72	84	82
PHD	35	35	35	38
Pseudo ARR-B	2	3	2	2
RB	1	1	1	1
Rcd1-like	2	2	2	2
SET	38	37	39	45
SNF2	32	31	34	37
SOH1	1	1	1	1
SWI/SNF-BAF60b	15	12	13	14
SWI/SNF-SWI3	4	4	4	3
TAZ	7	7	6	5
TRAF	26	28	24	29

**PKs**	**ET86**	**ET217**	**EC17**	**EG09**
AGC_MAST	2	2	2	2
AGC_NDR	5	5	5	5
AGC_PDK1	1	1	1	1
AGC_RSK-2	17	18	18	17
AGC-Pl	2	2	2	2
AGC_PKA-PKG	0	1	2	2
Aur	2	2	2	2
BUB	1	1	1	1
CAMK_AMPK	3	3	3	3
CAMK_CAMK1-DCAMKL	1	1	1	1
CAMK_CAMKL-CHK1	24	24	24	25
CAMK_CAMKL-LKB	1	1	1	1
CAMK_CDPK	36	38	39	41
CAMK_OST1L	9	10	9	8
CK1_CK1	3	4	3	5
CK1_CK1-Pl	10	11	13	12
CMGC_CDK-CCRK	1	1	1	1
CMGC_CDK-CDK7	1	1	1	1
CMGC_CDK-CDK8	1	1	1	1
CMGC_CDK-CRK7-CDK9	13	13	13	12
CMGC_CDK-PITSLRE	2	2	2	2
CMGC_CDK-Pl	3	3	4	4
CMGC_CK2	3	3	3	2

CMGC_CLK	3	3	3	3
CMGC_DYRK-PRP4	3	3	3	3
CMGC_DYRK-YAK	1	1	1	1
CMGC_GSK	7	7	7	7
CMGC_GSKL	1	1	1	1
CMGC_MAPK	16	15	13	18
CMGC_RCK	2	3	3	3
CMGC_SRPK	3	3	3	3
Group-Pl-2	1	1	0	1
Group-Pl-3	2	2	2	2
Group-Pl-4	2	2	2	2
IRE1	24	26	23	25
NAK	2	2	2	2
NEK	4	4	4	4
PEK_GCN2	2	2	2	2
RLK-Pelle_C-LEC	1	1	1	1
RLK-Pelle_CR4L	17	16	18	18
RLK-Pelle_CrRLK1L-1	40	37	35	36
RLK-Pelle_DLSV	318	329	327	349
RLK-Pelle_Extensin	8	7	7	7
RLK-Pelle_L-LEC	168	171	171	174
RLK-Pelle_LRK10L-2	110	118	133	139
RLK-Pelle_LRR-I-1	34	38	42	41
RLK-Pelle_LRR-I-2	2	2	2	2
RLK-Pelle_LRR-II	24	31	30	30
RLK-Pelle_LRR-III	58	56	56	53
RLK-Pelle_LRR-IV	8	8	8	7
RLK-Pelle_LRR-IX	9	11	8	10
RLK-Pelle_LRR-V	6	6	6	6
RLK-Pelle_LRR-VI-1	5	5	5	5
RLK-Pelle_LRR-VI-2	4	4	4	4
RLK-Pelle_LRR-VII-1	4	4	4	3
RLK-Pelle_LRR-VII-2	2	2	2	2
RLK-Pelle_LRR-VII-3	2	2	2	2
RLK-Pelle_LRR-VIII-1	15	17	10	18
RLK-Pelle_LRR-Xa	4	4	4	4
RLK-Pelle_LRR-Xb-1	15	12	15	14
RLK-Pelle_LRR-Xb-2	1	1	1	1
RLK-Pelle_LRR-XI-1	175	181	165	184
RLK-Pelle_LRR-XI-2	4	4	3	4
RLK-Pelle_LRR-XII-1	140	138	143	156
RLK-Pelle_LRR-XIIIa	4	4	3	3

RLK-Pelle_LRR-XIIIb	8	8	6	9
RLK-Pelle_LRR-XIV	2	2	2	2
RLK-Pelle_LRR-XV	2	2	2	3
RLK-Pelle_LysM	17	17	17	13
RLK-Pelle_PERK-1	35	33	35	36
RLK-Pelle_PERK-2	8	9	9	10
RLK-Pelle_RKF3	5	6	6	6
RLK-Pelle_RLCK-II	1	1	1	1
RLK-Pelle_RLCK-IV	4	4	4	4
RLK-Pelle_RLCK-IXa	2	2	2	2
RLK-Pelle_RLCK-IXb	30	28	31	27
RLK-Pelle_RLCK-Os	8	11	11	12
RLK-Pelle_RLCK-V	10	10	10	12
RLK-Pelle_RLCK-VI	14	14	13	13
RLK-Pelle_RLCK-VIIa-1	16	18	15	19
RLK-Pelle_RLCK-VIIa-2	55	55	55	58
RLK-Pelle_RLCK-VIIb	2	2	2	2
RLK-Pelle_RLCK-VIII	6	6	7	7
RLK-Pelle_RLCK-X	3	3	3	3
RLK-Pelle_RLCK-XI	4	4	3	3
RLK-Pelle_RLCK-XII-1	8	8	8	8
RLK-Pelle_RLCK-XIII	5	4	7	4
RLK-Pelle_RLCK-XV	3	3	3	3
RLK-Pelle_RLCK-XVI	1	1	1	0
RLK-Pelle_SD-2b	155	148	155	152
RLK-Pelle_Singleton	1	1	1	1
RLK-Pelle_URK-1	3	2	1	2
RLK-Pelle_URK-2	2	3	2	2
RLK-Pelle_WAK	125	125	131	128
RLK-Pelle_WAK_LRK10L-1	34	36	34	37
SCY1_SCYL1	0	1	1	1
SCY1_SCYL2	1	1	1	1
STE_STE11	29	33	32	31
STE_STE20-Fray	5	5	6	6
STE_STE20-Pl	1	1	1	1
STE_STE20-YSK	2	2	2	2
STE_STE7	5	6	6	7
STE_STE-Pl	1	1	1	1
TKL_CTR1-DRK-1	2	1	1	1
TKL_CTR1-DRK-2	9	11	11	10
TKL_Gdt	2	2	2	3
TKL-Pl-1	2	2	2	2

TKL-Pl-2	2	2	2	2
TKL-Pl-3	1	1	1	1
TKL-Pl-4	22	22	22	23
TKL-Pl-5	5	6	6	7
TKL-Pl-6	10	12	11	13
TKL-Pl-7	1	1	1	1
TKL-Pl-8	3	2	2	4
TLK	1	1	1	1
TTK	1	1	1	1
ULK_Fused	1	1	1	1
ULK_ULK4	1	1	1	1
WEE	2	2	2	2
WNK_NRBP	10	10	10	10
