# Supplementary material for: Chromosome-specific polymorphic SSR markers in tropical eucalypt species using low coverage whole genome sequences: systematic characterization and validation
Source: Genomics Inform. 2021 Sep 30;19(3):e33. doi: 10.5808/gi.21031 (PMC8510864; doi:10.5808/gi.21031)
Supplement: Supplemental Table 3. — SNPs and InDels predicted in clonal accessions of Eucalyptus (E. camaldulensis [EC17], E. tereticornis [ET217 and ET86], E. grandis [EG9]) [file gi-21031suppl3.pdf]

Supplementary Table 3. SNPs and InDels predicted in clonal accessions of *Eucalyptus* (*E. camaldulensis* [EC17], *E. tereticornis* [ET217 and ET86], *E. grandis* [EG9])

Pseudomolecules	EC17				ET217				ET86				EG09			
	SNPs	%	InDels	%	SNPs	%	InDels	%	SNPs	%	InDels	%	SNPs	%	InDels	%
Chr01	99,895	8.5	11,268	8.3	102,802	8.5	11,855	8.3	106,667	8.7	12,017	8.5	58,183	8	8,214	7.9
Chr02	111,498	9.5	12,779	9.5	115,554	9.6	13,730	9.6	114,239	9.3	13,414	9.5	67,481	9.3	9,799	9.4
Chr03	131,607	11.2	13,456	10	132,801	11	14,284	10	135,427	11	14,399	10.2	87,967	12.1	11,346	10.9
Chr04	85,080	7.3	9,940	7.4	90,466	7.5	10,689	7.5	89,245	7.3	10,334	7.3	52,945	7.3	7,768	7.4
Chr05	126,699	10.8	12,724	9.4	131,584	10.9	13,424	9.4	133,698	10.9	13,373	9.4	93,047	12.8	11,263	10.8
Chr06	110,946	9.5	15,006	11.1	115,958	9.6	15,541	10.9	113,958	9.3	15,030	10.6	62,440	8.6	10,349	9.9
Chr07	104,304	8.9	11,767	8.7	111,024	9.2	12,697	8.9	110,234	9	12,468	8.8	72,037	9.9	9,845	9.4
Chr08	151,996	13	16,830	12.5	151,013	12.5	17,473	12.3	155,535	12.7	17,672	12.5	88,576	12.2	12,946	12.4
Chr09	83,025	7.1	9,928	7.4	80,458	6.7	9,915	7	86,337	7	10,149	7.2	48,023	6.6	7,504	7.2
Chr10	77,618	6.6	10,012	7.4	81,971	6.8	10,655	7.5	86,558	7.1	10,856	7.7	42,221	5.8	7,051	6.7
Chr11	88,299	7.5	11,276	8.4	94,281	7.8	12,121	8.5	93,938	7.7	11,879	8.4	55,076	7.6	8,457	8.1
Total	1,170,967	100	134,986	100	1207,912	100	142,384	100	1,225,836	100	141,591	100	727,996	100	104,542	100
Mean	106,452	9.1	12,271	9.1	109,810	9.1	12,944	9.1	111,440	9.1	12,872	9.1	66,181	9.1	9,504	9.1
Maximum	151,996	13	16,830	12.5	151,013	12.5	17,473	12.3	155,535	12.7	17,672	12.5	93,047	12.8	12,946	12.4
Minimum	77,618	6.6	9,928	7.4	80,458	6.7	9,915	7	86,337	7	10,149	7.2	42,221	5.8	7,051	6.7

SNP, single nucleotide polymorphism.
