# Supplementary material for: Chromosome-specific polymorphic SSR markers in tropical eucalypt species using low coverage whole genome sequences: systematic characterization and validation
Source: Genomics Inform. 2021 Sep 30;19(3):e33. doi: 10.5808/gi.21031 (PMC8510864; doi:10.5808/gi.21031)
Supplement: Supplemental Table 4. — Top 15 types of simple sequence repeat motifs in clonal accessions of Eucalyptus having 1% and above distribution (E. camaldulensis (EC17), E. tereticornis (ET217 and ET86), E. grandis (EG9) [file gi-21031suppl4.pdf]

Supplementary Table 4. Top 15 types of simple sequence repeat motifs in clonal accessions of *Eucalyptus* having 1% and above distribution (*E. camaldulensis* (EC17), *E. tereticornis* (ET217 and ET86), *E. grandis* (EG9)

Rank	Grouped_Motif	EC17		ET217		ET86		EG09	
		Total	%	Total	%	Total	%	Total	%
1	AG/GA/CT/TC	42,708	45.01	42,669	44.74	42,731	44.78	42,633	44.24
2	AT/TA	10,998	11.59	11,322	11.87	11,246	11.79	11,583	12.02
3	AAG/GAA/TTC/CTT	6,393	6.74	6,459	6.77	6,491	6.80	6,552	6.80
4	AAT/TTA/TAA/ATT	3,990	4.20	4,062	4.26	4,024	4.22	4,045	4.20
5	AC/CA/TG/GT	3,061	3.23	3,023	3.17	3,021	3.17	3,046	3.16
6	AAAT/ATTT	2,268	2.39	2,293	2.40	2,306	2.42	2,220	2.30
7	AGA/TCT	2,126	2.24	2,136	2.24	2,162	2.27	2,158	2.24
8	CCG/GGC/GCC/CGG	2,069	2.18	2,109	2.21	2,109	2.21	2,182	2.26
9	AGG/TCC/GGA/CCT	2,066	2.18	2,105	2.21	2,092	2.19	2,115	2.19
10	CTC/GAG	1,362	1.44	1,361	1.43	1,368	1.43	1,377	1.43
11	AAAAT/ATTTT	1,312	1.38	1,267	1.33	1,312	1.37	1,237	1.28
12	ATG/TAC/GTA/CAT	1,095	1.15	1,133	1.19	1,094	1.15	1,115	1.16
13	ATC/TAG/CTA/GAT	1,059	1.12	1,073	1.13	1,091	1.14	1,095	1.14
14	ATA/TAT	1,003	1.06	1,004	1.05	1,004	1.05	1,050	1.09
15	AAAAG/CTTTT	1,001	1.05	978	1.03	964	1.01	1,008	1.05
