# Supplementary material for: Chromosome-specific polymorphic SSR markers in tropical eucalypt species using low coverage whole genome sequences: systematic characterization and validation
Source: Genomics Inform. 2021 Sep 30;19(3):e33. doi: 10.5808/gi.21031 (PMC8510864; doi:10.5808/gi.21031)
Supplement: Supplemental Table 5. — Length distribution of top 15 types of simple sequence repeat motifs in clonal accessions of Eucalyptus (E. camaldulensis (EC17), E. tereticornis (ET217 and ET86), E. grandis (EG9) [file gi-21031suppl5.pdf]

Supplementary Table 5. Length distribution of top 15 types of simple sequence repeat motifs in clonal accessions of *Eucalyptus* (*E. camaldulensis* (EC17), *E. tereticornis* (ET217 and ET86), *E. grandis* (EG9)

**Repeats**	**EC17**			**ET217**			**ET86**			**EG9**		
	**<20 bp**	**20–40 bp**	**>40 bp**	**<20 bp**	**20–40 bp**	**>40 bp**	**<20 bp**	**20–40 bp**	**>40 bp**	**<20 bp**	**20–40 bp**	**>40 bp**
AT/TA	9,522	1,432	44	9,922	1,355	45	9,832	1,371	43	10,276	1,264	43
AG/GA/CT/TC	37,321	5,099	288	37,472	4,907	290	37,466	4,975	290	37,465	4,870	298
CG/GC	143	0	0	152	0	0	156	0	0	172	0	0
AC/CA/TG/GT	2,718	340	3	2,711	309	3	2,708	309	4	2,730	312	4
AAC/TTG/CAA/GTT	561	2	0	3,515	487	60	3,460	502	62	3,473	512	60
AAT/TTA/TAA/ATT	3,417	514	59	2,115	19	2	2,144	16	2	2,141	15	2
AGA/TCT	2,105	19	2	6,407	52	0	6,437	54	0	6,486	66	0
AAG/GAA/TTC/CTT	6,339	54	0	572	1	0	545	2	0	563	3	0
ACC/TGG/CCA/GGT	564	0	0	574	0	0	567	0	0	572	0	0
ACA/TGT	121	1	0	130	0	0	108	0	0	109	1	0
ACG/TGC/GCA/CGT	423	0	0	400	0	0	404	0	0	434	0	0
ACT/TGA/TCA/AGT	470	2	0	508	4	0	507	2	0	487	3	0
AGC/TCG/CGA/GCT	537	0	0	548	0	0	553	0	0	587	0	0
AGG/TCC/GGA/CCT	2,066	0	0	2,105	0	0	2,092	0	0	2,115	0	0
ATA/TAT	822	163	18	814	173	17	820	168	16	838	155	15
