# Supplementary material for: Chromosome-specific polymorphic SSR markers in tropical eucalypt species using low coverage whole genome sequences: systematic characterization and validation
Source: Genomics Inform. 2021 Sep 30;19(3):e33. doi: 10.5808/gi.21031 (PMC8510864; doi:10.5808/gi.21031)
Supplement: Supplemental Table 6. — SSR marker information and details on primer pairs synthesised for validation in the cross E. tereticornis ET86 x E. grandis EG9. [file gi-21031suppl6.pdf]

Supplementary Table 6. SSR marker information and details on primer pairs synthesised for validation in the cross *E. tereticornis* ET86 x *E. grandis* EG9.

S.No	SSR Code	Forward Primer	Forward Tm	Reverse Primer	Reverse Tm	SSR Motif	Repeat Type	No. of Repeats	Product size	No of alleles in ET86	No of alleles in EG9	Status
1	GWET11	TTGACTGATTTG GCAATGGA	60.049	CACCGCATGGAGGTT CTTAT	59.955	CT	p2	7	132	130, 134	132, 138	Polymorphic
2	GWET12	GTATCTCTCGCC TCCAATCG	59.797	TGGCTAACGGAAAAT GTTGA	59.157	AAG	p3	6	249	249, 249	249, 249	Monomorphic
3	GWET13	TCTCCCCAAGAA GAAGACGA	59.92	ACCGGAAACCATTGA CTCTG	59.966	TC	p2	9	162	168, 168	164, 164	Polymorphic
4	GWET14	TTTTACCAAAGG ACGCAACC	59.975	TAAGCGTGGATGTTC GTTCA	60.257	ACAT	p4	10	251	250, 258	250, 254	Polymorphic
5	GWET15	CCTCAGTGATGA ATGCTTGG	59.241	ACAAACGCTGCCTTA ATGGT	59.637	TA	p2	10	365	-	-	No Amplification
6	GWET16	ACCGATCTCCAC AAAACAGC	60.119	GGCACCAAGTTGTCA TCAAA	59.547	AAT	p3	16	205	205, 208	208, 212	Polymorphic
7	GWET17	GCCGAAAAAGC CCAACAACT	59.896	GCGAGTTTCGACTGG AGGAA	60.039	AGA	p3	5	174	177, 183	175, 182	Polymorphic
8	GWET21	ATCTGATGGATG CGGTTGA	60.023	GCAGGAGCCGGAAA TACATA	60.06	GGA	p3	5	273	275, 275	275, 275	Monomorphic
9	GWET22	CCGGTTTGGTTT CAGTCAAT	59.83	CGATGAAAGCACCAA AGACA	59.84	TGC	p3	7	265	260, 260	264, 264	Polymorphic
10	GWET23	TCGTTGAAAGAT CCCTGGAG	60.187	ATTGAGGCCAAATAC CCACA	60.192	GCGA	p4	5	245	245, 250	250, 250	Polymorphic
11	GWET24	CTCTCGGCTTCC TCTTCACC	59.825	GAAAGGACAGGGCTT GCTGA	60.251	CAG	p3	6	131	130, 138	132, 138	Polymorphic
12	GWET25	GACTATAAAAC GGGGCGTGA	59.96	ATGGCGCTATCACAG AATCC	60.066	AG	p2	6	209	-	-	No Amplification
13	GWET26	GATGGCCACCA GGCTATATG	60.318	CAACTCCATCGAGGG GTAAA	59.926	AT	p2	8	381	-	-	No Amplification
14	GWET27	GAAGGGGAGTT CGAAGGAAG	60.183	TCCCAACCCTTGAAA TTCTG	59.903	TAGT	p4	5	320	319, 320	326, 320	Polymorphic
15	GWET31	CCTCACTGAGGG TTGGTGAC	59.965	TGCATTTAACACCGT GCTCA	58.401	GAA	p3	18	233	-	-	No Amplification
16	GWET32	TGCTGCTGCTAG TTGCTGAA	60.25	GTCAGCTTCCATCCC CGAAA	60.035	GA	p2	14	183	198, 210	194, 204	Polymorphic
17	GWET33	TGGTGTCATTGC GGAGAACA	59.892	GAACATCGGAAGGTC GGAGG	60.179	CCT	p3	5	142	147, 147	147, 147	Monomorphic
18	GWET41	TGCGAGCAGGG AAATTTTAT	59.682	ACAAGCACCCATCTT TCAGC	60.263	TTTA	p4	7	224	220, 224	224, 228	Polymorphic
19	GWET42	TTTCGACCCACT ATGGTTGC	60.894	AAGTTTCCGTTCGAC CACAC	60.012	CT	p2	8	178	178, 180	184, 186	Polymorphic

20	GWET43	CGGCTAACGAAGAGAACGAC	60.015	GCTCGTCACAAATTCAGCAA	59.995	GAA	p3	6	209	209, 224	213, 213	Polymorphic
21	GWET44	TTTGGTTTTAGGGCGAGAGA	59.817	GGTTCTTGTTCGGTGTTCGT	60.012	AG	p2	7	255	254, 256	256, 258	Polymorphic
22	GWET45	GGGGAAAGAATTCACAGCAA	60.051	GTGGGCTTCTTCGACTTGAG	59.989	CGCT	p4	5	285	289, 289	285, 289	Polymorphic
23	GWET46	GGGCTCTAAAACCCTGGAAT	59.42	ATGACTCTGCTGCTTCCACA	59.577	CGG	p3	8	198	200, 206,	203, 206	Polymorphic
24	GWET47	GCACACCCTACCAGGAGAGA	60.261	GTTTGGCAAGCTCATCGAA	59.94	TCT	p3	11	178	-	-	No Amplification
25	GWET51	AGCTCAAGCAAGCACCTCTC	59.898	TTGACCGAGTTGTCGAAGTG	59.873	CT	p2	10	212	201, 212	211, 212	Polymorphic
26	GWET52	GCGCCTGCGACAGATAGTAG	61.091	CCCTCTTGGCCATTGATTTA	59.894	AG	p2	9	282	280, 300	264, 280	Polymorphic
27	GWET53	CGGGGAGAAGCTGGAGTAG	59.951	AAAATCGCTGACCAGAAAGC	59.457	CGG	p3	6	325	324, 325	331, 325	Polymorphic
28	GWET54	GGGTTCCCATATACCACACC	58.838	CATTCTCGGGCACATTCTTT	60.074	AAT	p3	6	327	-	-	No Amplification
29	GWET55	GGGCGTCTGACAACAAGAGT	60.307	TTGAACGGACCTGGAGTTTC	60.088	GA	p2	8	303	300, 304	310, 312	Polymorphic
30	GWET56	GAGTGGAAAGACCATGCACA	59.682	GTTTTGGGAGGAGAACATGC	59.532	TC	p2	14	223	224, 224	224, 224	Monomorphic
31	GWET57	TAACTTTTGGGCTGGATTGG	59.931	TCGGTTAGGTTTTGGTTTCG	59.968	TC	p2	11	196	198, 202	200, 202	Polymorphic
32	GWET61	GCATACGTGTCGCATGTGTC	59.975	AGAAGTGGGACATCAGCTGC	60.036	GAA	p3	17	212	-	-	No Amplification
33	GWET62	GCTCCTCTCCCTCTCTCCAT	59.812	GACATGCAACGATCTCACGC	59.974	CT	p2	14	121	120, 120	104, 112	Polymorphic
34	GWET63	CGTTTGCCTCTGCTTGCTTT	59.969	CCAGACGCCACAACAAACAG	59.971	CT	p2	11	174	169, 175	168, 174	Polymorphic
35	GWET71	GACTGCTATCGCACCACCAT	60.179	ACAGAACAGGGCACAAGCAT	60.179	CA	p2	6	168	168, 168	168, 177	Polymorphic
36	GWET72	CCTCCGTTGACTCGCTCTTC	60.457	TCGAGGAAGAGGTGGGAGAC	60.324	GCG	p3	5	127	125, 125	125, 125	Monomorphic
37	GWET73	TGCAATGCTTGACTTGTGATGA	59.114	TACAAACTCCAGCACTCGGG	59.68	TC	p2	6	137	132, 132	132, 132	Monomorphic
38	GWET81	CGAACGACATCATTCCACAG	60.112	TCGCGATCTCTCGTAGTGAA	59.697	CG	p2	6	168	164, 164	164, 164	Monomorphic
39	GWET82	TGAACCGCATCGTTCTGTTA	60.257	ATTGAAACGGTGTGGAATGG	60.615	AC	p2	7	284	279, 284	283, 284	Polymorphic
40	GWET83	AGGTGTGCAAGAAAGTCACCA	60.065	CCAGCTTGGAGGTCGGAAAT	60.035	AAG	p3	7	205	202, 206	200, 200	Polymorphic
41	GWET84	AATCTCCCCGTCCTGAATCT	59.896	CCAAGCTCAAATCCAAGCTC	59.955	AAGGG	p4	6	225	-	-	No Amplification

42	GWET85	GGAAGCCATGTTCTCCATGT	59.934	TGTGGACATCCAAAACTCCA	59.935	CT	p2	8	266	275, 275	275, 275	Monomorphic
43	GWET86	GAATTCGTGACTCCGGCTAA	60.214	TGTCGCTTCGAGTGTGTTTC	60.032	GA	p2	18	196	196, 202	202, 202	Polymorphic
44	GWET87	ACGTTTTCAAAGCCCAAATG	59.975	CCCACTGATGACGGAAGATT	59.927	AT	p2	7	212	-	-	No Amplification
45	GWET91	GCCCACCACTTGTTCTGGTT	60.755	CGAGAGAGTTTAGGCGCTCA	59.548	TTC	p3	5	173	170, 178	174, 181	Polymorphic
46	GWET92	GGCATACAGAGACACCCCAC	60.108	TGTCGCACAAGGAATATAAATAGTCG	59.58	ATC	p3	7	146	142, 148	148, 152	Polymorphic
47	GWET93	ATGGGAAGATGAGTCGGTGC	59.821	ATTCAACGTGCCAGCAATGT	59.039	CT	p2	15	146	148, 156	143, 152	Polymorphic
48	GWET101	ATTGTTGAGGAGGGGACCTT	59.795	GTGATGGAGGTCAACACACG	60.005	GAA	p3	7	268	-	-	No Amplification
49	GWET102	CACCACAGCTCCCCATTTCT	59.961	CGGTCGAGTGTTAGCCAGTT	60.039	TC	p2	18	116	116, 118	116, 120	Polymorphic
50	GWET103	GAATAGGGCCCAAGCATGTA	59.923	CAAACCGACCTGTAGGGAGA	60.103	AT	p2	5	215	214, 215	224, 215	Polymorphic
51	GWET104	AAATATAGGCGAGCGGGTTC	60.428	CCTGTCGCATTTTGGTTTCT	60.11	CCA	p3	5	201	200, 201	206, 201	Polymorphic
52	GWET105	CGGTGGTCATGTTAGTGGAA	59.415	CCACTTCAAAAGAGGCTTCG	59.986	GAG	p3	5	172	171, 172	160, 172	Polymorphic
53	GWET106	AGCTCAGAGGGTGGACATTC	59.258	AAGAGGCGTGTTTCGTGTCT	59.914	CCG	p3	5	301	298, 298	298, 298	Monomorphic
54	GWET107	ATTAAATTCCCATCGGCACA	60.153	GTGTCGAGAACCCCTTTTCA	60.088	CT	p2	8	258	250, 250	250, 250	Monomorphic
55	GWET108	GCGACAGCTTATCGTCCTTC	59.985	CCAACAACGAACAAAACGTG	60.043	AG	p2	8	226	-	-	No Amplification
56	GWET111	GATAGTCTCTCGCCTGCTGC	60.319	TGACCCAAACAATGCTCAGC	59.039	GT	p2	8	178	175, 175	175, 175	Monomorphic
57	GWET112	CAAGGTGTGAGCCTCAGGAG	60.037	CCTTGCAGAGGCTTAATGGAG	58.704	AG	p2	8	153	-	-	No Amplification
58	GWET113	CTATGGGGAGCCGTTGTCTC	59.895	TGACAAGCCTTGGTACTGGC	60.251	AGA	p3	10	127	122, 128	124, 124	Polymorphic
